# Global burden of multiple myeloma(1990–2021) and projections up to 2035: a methodical analysis leveraging the global burden of disease 2021 study and Mendelian randomization

**DOI:** 10.1186/s43046-026-00377-4

**Published:** 2026-07-09

**Authors:** Run Qu, Xin Zhang, Jing Zou, Jinghua Ning, Yanhong Zhao, Yi Liang, Yuzhe Zhang

**Affiliations:** 1 College of Basic Medical Sciences, Dali University, Dali, China; 2 Department of Respiratory Medicine, The First Affiliated Hospital, Dali University, Dali, China; 3 Princess Margaret Cancer Centre, University Health Network, TMDT-MaRS Centre, Toronto, ON M5G 1L7 Canada; 4 Yunnan Provincial Key Laboratory of Insect Medicine Research and Development, Dali, China

**Keywords:** Multiple Myeloma, Disease Burden, Mendelian randomization, Socio-Demographic Index, Projection

## Abstract

**Objective:**

Multiple myeloma (MM), being the second-most frequently occurring hematologic malignancy, is comprehensively assessed for its global disease burden and risk factors using the Global Burden of Disease (GBD) database and Mendelian Randomization (MR).

**Methods:**

This research utilized data from the GBD 2021 database to extensively investigate age-standardized incidence rates, mortality rates, as well as Disability - Adjusted Life Years (DALYs) linked to MM. We evaluated the impact of various geographical areas, age brackets, genders, risk factors, and Socio-Demographic Index (SDI) on the disease burden of MM. Additionally, Joinpoint regression was employed by us to analyze the temporal trends of this burden. We integrated GBD data with a two-sample MR method for causal inference to further investigate causal relationships between risk factors and MM. Finally, we applied Bayesian-Age-Period-Cohort (BAPC) models to predict future trends in MM’s disease burden by using GBD data as a foundation.

**Results:**

In 2021, the global incidence of MM was 148,755 cases, with 116,359 deaths and a total of 2,595,595 DALYs. The global age-standardized rates of MM were 1.74 (incidence), 1.37 (mortality), and 30.00 (DALYs) per 100,000 population, with higher rates observed in high-income areas. From 1990 to 2021, the annual average percentage change (AAPC) in global MM incidence was 0.54%, mortality was 0.20%, and DALYs was 0.18%. The MM burden associated with high BMI was concentrated within high-income areas such as those in Australasia and North America, while South Asia and Southeast Asia exhibited the lowest proportional burden. To further validate the causal link between BMI and MM, we conducted a two-sample MR.

**Conclusion:**

Over 30 years, the worldwide burden of MM has shown a significant rise. High-income countries show slowing trends due to improved healthcare, while low/middle-income regions face rising burdens from underdiagnosis and limited access. We demonstrate a positive association between elevated BMI and increased MM risk by using MR, underscoring the imperative for obesity control in high-income nations to alleviate the MM disease burden. This study offers a basis for optimizing global health policies, improving early diagnosis, and resource allocation against the ongoing threat of MM.

**Supplementary Information:**

The online version contains supplementary material available at 10.1186/s43046-026-00377-4.

## Introduction

Multiple myeloma (MM) is a malignancy involving plasma cells. While it can be treated, there is no present cure. It is marked by the uncontrolled and mutated growth of plasma cells within the bone marrow, which leads to damage specific to various organs [1]. MM is the second-most frequently occurring hematologic malignancy, following non-Hodgkin lymphoma and leukemia, accounting for approximately 14% of all new cases of hematologic cancers [2]. Moreover, significant variations can be observed in the incidence and mortality rates of MM between sexes [3]. A thorough comprehension of the global epidemiological features of MM, including gender disparities, is critical for formulating efficient public health strategies.

Although the precise etiology of MM remains incompletely understood, accumulating evidence has demonstrated that environmental and occupational exposures, antigenic stimulation, and genetic factors play essential parts in the onset and progression of this disease [4]. Several risk factors, including obesity, gender, age, and chronic inflammation, have a direct link to MM [5]. Notably, Obesity plays a significant part in increasing the probability of getting MM. Also, an increased body mass index (BMI) is strongly associated with MM disease progression [6]. Previous meta-analyses have indicated that BMI is positively linked with MM risk [7]. Therefore, lowering BMI can be an effective measure to prevent MM. Leveraging the Global Burden of Disease (GBD) database, the investigation identified high body mass index (BMI) as a key modifiable risk factor. This significant finding not only provides crucial insights for disease prevention but also informs evidence-based public health intervention strategies.

The GBD 2021 data is used to explore the epidemiology of a wide range of diseases. It provides crucial indicators like disease prevalence, incidence, mortality, and DALYs. As an important tool for assessing the global disease burden linked to MM, the GBD framework presents epidemiological data and trends across countries, regions, and SDI subregions [8]. Given the disparities in global economic development, this study specifically focuses on the variations in the MM disease burden across regions with different SDI levels. By examining these variations within different contexts of economic development levels, this research aims to offer a scientific basis for mitigating global disparities in disease burden [9].

In contrast to previous studies that have predominantly concentrated on specific countries or regions [10, 11], this investigation represents the first comprehensive global analysis concerning the disease burden posed by MM. It systematically examines the gender and regional differences in MM incidence, mortality, and DALYs over an extensive period, from 1990 to 2021. Additionally, this research integrates worldwide data while offering predictions on future disease trends extending up to 2035. This study not only fills significant gaps in the existing literature but also serves as an invaluable reference for formulating public health strategies.

This study’s primary aim was to reveal the global epidemiological characteristics of multiple myeloma (MM) and to clarify its disease burden and changing trends under the influence of different countries, regions, levels of economic development, and risk factors (such as high BMI). On this foundation, we employed a two-sample MR method, using genetic variation as instrumental variables (IVs), to evaluate the causal connection between high BMI and MM. This strategy effectively circumvented the interference of confounding factors like the environment and lifestyle, thereby minimizing the bias inherent in traditional observational studies. By integrating epidemiological characteristics with causal inference findings, we have conducted a comprehensive disease burden analysis that provides an evidence-based foundation for developing targeted public health measures, thus effectively mitigating the global health threat posed by MM.

## Methods

### Data source

This study is founded on the data of the GBD 2021, which integrates information from 100,983 data sources such as vital registration systems, verbal autopsy records, and population censuses, covering 288 causes of death concerning both incidence and mortality. To guarantee the strength and dependability of the data analysis, GBD 2021 employs advanced statistical modeling techniques, notably the Cause of Death Ensemble Model (CODEm). In order to address the variability in the quality of epidemiological data across different regions, GBD 2021 estimates missing data through a combination of predictive modeling, data sharing, and expert advice [12].

For this study, data regarding MM, including incidence, mortality, and DALYs, along with their respective ASRs, were obtained from the Global Health Data Exchange (GHDx) (https://vizhub.healthdata.org/gbd-results/) [8]. Special attention was given to the disease burden data for multiple myeloma across various SDI regions, enabling a comparative analysis of regional disparities.

As a composite index, the SDI mirrors the state of social development at the national scale. It is calculated based on three core variables: (1) the total fertility rate for individuals below 15 years old; (2) the average years of schooling for individuals aged 15 and older; and (3) the distribution of per capita income, which together provide a nuanced representation of social development across different regions. The SDI ranges from 0 to 1, with increasing values signifying a more elevated socioeconomic status. The Global Burden of Disease Study 2021 classifies 204 countries and regions into five distinct SDI categories based on the SDI: low, low-middle, middle, high-middle, and high SDI [13].

### Trend analysis: joinpoint regression model

This research used the Joinpoint regression model to analyze the time-related trends in the MM burden between 1990 and 2021. Joinpoint is a segmented regression technique that identifies inflection points in a temporal trend by fitting a series of log-linear regression models, illustrated through the following equation:

$$\mathrm{ln}\left(\mathrm{y}\right)=\beta\cdot\mathrm{x}+\mathrm{constant}$$

In this context, y stands for the ASRs, x represents the temporal variable, and β is the regression coefficient of ln(y) in the regression model, which includes the time variable [14]. Using the model, we calculated the Annual Percentage Change (APC) and the Average Annual Percentage Change (AAPC) both globally and for each SDI region, reporting the corresponding 95% confidence intervals (95% CI) [15]. The formulas are as follows:

$$\mathrm{APC}=\left(\mathrm{e}^\beta-1\right)\times\mathrm{100}{\%}$$

The AAPC is calculated as the weighted mean of the APCs across the analyzed timeframe [14]. Additionally, to further assess MM temporal trends, the EAPC was derived through the following modeling approach:

$$\mathrm{ln}\left(\mathrm{ASRs}\right)=\alpha+\beta\;\left(\text{calendar year}\right)+\epsilon$$

The 95% confidence interval (CI) for EAPC is calculated using the following formula [16]:

$$\mathrm{EAPC}=100\times\left(\mathrm{e}^\beta-1\right)$$

If the EAPC is greater than 0 and the p-value is less than 0.05, it implies an upward trend. Conversely, when the EAPC is less than or equal to 0 or a p-value greater than or equal to 0.05, it suggests a decreasing trend [17–19]. When the 95% CI contains 0, the difference is not regarded as statistically significant [20]. In country-level analyses, if both the upper and lower bounds of the 95% CI are either positive or negative, it indicates that the corresponding age-standardized rate (ASR) is significantly trending upward or downward. On the other hand, when the CI encompasses zero, it suggests that the observed trend lacks statistical significance.

### SDI-related correlation analysis

This study employed smoothed curves regression to investigate socioeconomic disparities in multiple myeloma (MM) epidemiology across 21 GBD regions and 204 countries, analyzing three key age-standardized metrics: Incidence rates (ASIR), Mortality rates (ASMR), and Disability-adjusted life years rates (AS-DALYsR). This analysis was conducted using the Locally Weighted Scatterplot Smoothing (Lowess) method, a smoothed line model. Additionally, Correlation coefficients and their significance (*P* < 0.05) for ASR and SDI were determined through Spearman’s correlation analysis. The methodological framework of this study integrates both trend analysis and SDI correlation analysis, providing a comprehensive approach to elucidating the variations in the worldwide MM burden and identifying its contributing factors.

### Risk factor

The GBD 2021 report recognizes high BMI as the sole risk factor associated with MM. Drawing upon GBD 2021 datasets, we systematically and comprehensively examined the ratio of the MM disease burden caused by high BMI in all GBD regions and five SDI regions. The analysis highlights regional variations in the MM burden related to high BMI and provides data to inform relevant prevention and intervention strategies.

### Mendelian randomization analysis

This investigation combined GBD datasets with MR approaches to examine MM risk factors and their potential causal relationships. The GBD 2021 report suggests that BMI serves as a risk factor for MM [21]. To further verify the causal association between BMI and MM, the two-sample MR method was used to analyze the causal effects of IVs in GWAS. BMI data [22] were obtained from the European Bioinformatics Institute GWAS database, including 224,849 cases and 456,426 controls of European ancestry. The MM data were derived from the FinnGen database (https://www.finngen.fi/en/access_results), which included 585 cases and 287,129 controls. In this study, instrumental variables were selected through rigorous quality control to fulfill the 3 assumptions underlying MR analysis and to guarantee the stability and dependability of MR analysis [23]. The selection process consisted of three sequential steps: (Ⅰ) Obtained SNPs associated with BMI (*P* < 5 × 10^− 8^). (Ⅱ) Eliminate linkage disequilibrium (LD) among SNPs, because strong LD may result in inaccurate outcomes (r^2^ < 0.001, Kd = 10,000 kb). (Ⅲ) Exclude SNPs associated with MM. For all IVs, it is necessary to compute their F-statistics to evaluate the strength of these instruments. When the F-value exceeds 10, it means that the chosen IVs possess sufficient strength to achieve adequate estimation results in the subsequent MR analysis, and there is no weak instrument variable bias. Any IVs failing to meet the F > 10 criterion will be eliminated.

The PhenoScanner database (http://www.phenoscanner.medschl.cam.ac.uk/) was employed to examine linkages between IVs and potential confounders. IVs demonstrating associations with confounding factors at a significance level of *P* < 5 × 10⁻⁶ were systematically excluded from our analysis.

For the genetic causal effect of BMI on MM, the primary method adopted was the Inverse Variance Weighted (IVW) approach. Additionally, MR Egger, weighted median, simple mode, and weighted mode were utilized as complementary methods for MR analysis. We evaluated instrumental variables heterogeneity using Cochran’s Q statistic. When the P-values exceeded 0.05, this signified that there was no substantial heterogeneity; conversely, it was the opposite. Additionally, the absence of horizontal pleiotropy was determined when the P-values of the MR-Egger intercept exceeded 0.05. Finally, we performed the leave-one-out method to determine whether any individual SNP affected the BMI-MM association.

This MR study utilized GWAS summary statistics or shared datasets. Therefore, there was no need for a separate ethical statement or consent form.

### Bayesian age-period-cohort model prediction

In this study, we employed the Bayesian Age-Period-Cohort (BAPC) model, a method based on Integrated Nested Laplace Approximations (INLA). We assumed that the available data conformed to the BAPC model, which is widely used to predict future trends in chronic diseases, such as cancer [24, 25]. This study developed a BAPC model using the 1990–2021 data on MM from the GBD 2021 study, leveraging the “BAPC” and “INLA” packages within the R environment. We employed a BAPC model to forecast global and regional epidemiological trends in MM burden through 2035, generating evidence to inform strategic public health planning and resource allocation.

### Statistics

The study systematically conducted the MM disease burden. This was achieved through an analysis of its incidence, mortality, and DALYs. We expressed disease burden measures as ASRs per 100,000, accompanied by 95% Uncertainty Interval (95% UI). We also analyzed trends in the quantity of cases over time. All data were processed using appropriate statistical models, with α = 0.05 as the significance threshold. All statistical analyses were executed by means of the R software (version 4.4.1).

## Results

### MM disease burden at global, regional, and national levels

In 2021, the global incidence of MM was 148,755 cases (95% UI: 131,780–162,049), with an ASIR of 1.74 per 100,000 population (95% UI: 1.57–1.89). The global mortality of MM was 116,359 cases (95% UI: 103,078–128,470), with an ASMR of 1.37 per 100,000 population (95% UI: 1.22–1.52). The total number of DALYs was 2,595,595 (95% UI: 2,270,484–2,889,969), with an AS-DALYsR of 30.00 per 100,000 population (95% UI: 26.22–33.37) (Table 1).

**Table 1:** Disease burden of multiple myeloma

	Incidence (95% UI)				Deaths (95% UI)			DALYs (95% UI)		
	Counts (2021)	ASR			Counts (2021)	ASR		Counts (2021)	ASR	
		2021		1990–2021		2021	1990–2021		2021	1990–2021
Global	**148**,**755**		**1.74**	**0.18**	**116**,**359**	**1.37**	**0.06**	**2**,**595595**	**30.00**	**0.06**
	**(131**,**780 − 162**,**049)**		**(1.57–1.89)**	**(0.08 to 0.28)**	**(103**,**078–128**,**470)**	**(1.22–1.52)**	**(-0.03 to 0.16)**	**(227**,**048**,**4-288**,**996**,**9)**	**(26.22–33.37)**	**(-0.04 to 0.16)**
High SDI	68,288		3.16	0.06	51,434	2.28	-0.09	976,932	47.33	-0.15
	(61,342 − 72,525)		(2.87–3.34)	(0.001 to 0.11)	(45,704 − 54,830)	(2.05–2.41)	(− 0.14 to -0.05)	(896,756-1,033,833)	(44.00-49.82)	(− 0.18 to -0.11)
High-middle SDI	34,786		1.75	0.38	25,451	1.28	0.21	583,311	29.65	0.19
	(30,245 − 38,625)		(1.52–1.95)	(0.19 to 0.52)	(22,131 − 28,174)	(1.12–1.43)	(0.06 to 0.35)	(504,141–652,354)	(25.53–33.21)	(0.008 to 0.33)
Low SDI	3,801		0.77	0.36	3,648	0.73	0.33	99,828	18.43	0.29
	(2,523-5,131)		(0.51–1.02)	(0.06 to 1.17)	(2,427-4,903)	(0.47–0.98)	(0.05 to 1.08)	(66,163 − 136,137)	(12.25–24.91)	(− 0.001 to 1.06)
Low-middle SDI	13,201		0.92	0.72	12,282	0.89	0.62	323,358	21.51	0.59
	(11,293 − 18,483)		(0.79–1.30)	(0.38 to 1.52)	(10,526 − 17,215)	(0.76–1.24)	(0.30 to 1.38)	(274,506 − 449,769)	(18.34–29.96)	(0.28 to 1.35)
Middle SDI	28,498		1.05	0.16	23,404	0.88	0.79	609,118	21.85	0.78
	(22,906 − 33,492)		(0.84–1.23)	(− 0.07 to 0.40)	(18,799 − 27,500)	(0.71–1.04)	(0.38 to 1.15)	(487,413–714,664)	(17.50-25.56)	(0.37 to 1.16)
Latin America and Caribbean										
Andean Latin America	1,061		1.80	0.57	883	1.51	0.34	22,215	36.98	0.33
	(824-1,379)		(1.39–2.33)	(0.19 to 1.02)	(692-1,147)	(1.19–1.96)	(0.03 to 0.71)	(17,273 − 29,040)	(28.79–48.19)	(0.02 to 0.71)
Caribbean	1,711		3.17	0.33	1,109	2.05	0.12	26,898	49.86	0.16
	(1,464-1,960)		(2.71–3.63)	(0.15 to 0.51)	(957-1,256)	(1.77–2.32)	(− 0.01 to 0.27)	(23,074 − 30,706)	(42.79–56.92)	(0.007 to 0.32)
Southern Latin America	2,033		2.33	0.05	1,675	1.54	-0.09	37,800	43.97	-0.11
	(1.875-2,190)		(2.15–2.50)	(− 0.05 to 0.14)	(1,544-1,791)	(1.37–1.68)	(− 0.17 to -0.02)	(35,378 − 40,287)	(41.24–46.90)	(− 0.19 to -0.04)
Tropical Latin America	5,412		2.09	-0.07	4,585	1.79	0.47	113,479	43.43	0.39
	(5,051 − 5,693)		(1.95–2.21)	(− 0.11 to -0.02)	(4,254-4,822)	(1.66–1.89)	(0.40 to 0.54)	(107,328 − 118,279)	(41.00-45.28)	(0.33 to 0.45)
Central Europe, eastern Europe, and central Asia										
Central Asia	440		0.50	0.82	386	0.45	0.75	11,936	13.06	0.67
	(392–492)		(0.45–0.56)	(0.56 to 1.11)	(344–434)	(0.41–0.51)	(0.49 to 1.03)	(10,635 − 13,424)	(11.63–14.64)	(0.42 to 0.95)
Central Europe	4,952		2.21	0.54	4,652	1.31	0.51	95,122	43.87	0.32
	(4,505-5,371)		(2.01–2.40)	(0.41 to 0.67)	(4,295-5,042)	(1.21–1.42)	(0.39 to 0.66)	(86,993 − 103,200)	(40.13–47.69)	(0.21 to 0.43)
Eastern Europe	6,169		1.76	0.71	74,966	21.00	-0.12	118,762	34.51	0.42
	(5,703-6,689)		(1.63–1.90)	(0.56 to 0.85)	(68,115 − 81,565)	(19.11–22.87)	( -0.32 to 0.09)	(109,042–128,799)	(31.74–37.42)	(0.30 to 0.56)
High-income Australasia	2,991		5.48	0.32	1,656	2.89	0.02	32,239	60.65	-0.06
	(2,604-3,385)		(4.77–6.21)	(0.13 to 0.52)	(1,438-1,854)	(2.52–3.23)	(− 0.10 to 0.14)	(28,598 − 35,867)	(54.09–67.37)	(− 0.15 to 0.08)
High-income										
High-income North America	20,898		3.10	-0.07	197,315	28.92	-0.42	374,036	57.28	-0.22
	(19,023 − 22,011)		(2.83–3.26)	(-0.11 to -0.04)	(181,478 − 207,755)	(26.73–30.38)	( -0.44 to -0.36)	(348,987 − 390,777)	(53.80-59.76)	( -0.25 to -0.20)
High-income Asia Pacific	9,740		1.93	-0.03	11,618	21.71	-0.16	118,265	25.57	-0.24
	(8,163 − 10,907)		(1.66–2.16)	(-0.14 to 0.06)	(99,373 − 125,884)	(19.15–23.31)	( -0.23 to -0.11)	(101,403 − 130,303)	(22.47–27.98)	(− 0.33 to -0.18)
Western Europe	41,185		4.30	0.21	247,740	26.36	-0.26	492,170	53.56	-0.06
	(36,907 − 44,033)		(3.91–4.57)	(0.14 to 0.29)	(226,537 − 261,738)	(24.47–27.69)	(− 0.30 to -0.23)	(447,363–523,560)	(49.55–56.61)	(− 0.11 to -0.02)
North Africa and Middle East										
North Africa and Middle East	5,840		1.30	0.57	4,708	1.09	0.36	124,880	26.02	0.31
	(4,386-8,004)		(0.97–1.77)	(0.13 to 1.26)	(3,544-6,487)	(0.83–1.51)	(− 0.004 to 0.94)	(93,507 − 171,852)	(19.48–35.88)	( -0.06 to 0.87)
South Asia										
South Asia	15,905		1.09	0.73	14,790	1.04	0.62	383,531	24.96	0.58
	(12,551 − 21,561)		(0.86–1.47)	(0.31 to 1.84)	(11,658 − 20,033)	(0.83–1.41)	(0.23 to 1.65)	(302,576 − 511,789)	(19.65–33.42)	(0.19 to 1.62)
Sub-Saharan Africa										
Central Sub-Saharan Africa	239		0.44	0.20	5,078	9.48	-0.07	6,648	10.89	0.16
	(131–345)		(0.24–0.63)	(− 0.21 to 0.70)	(3,592-7,649)	(6.81–14.11)	(− 0.32 to 0.27)	(3,617-9,691)	(5.93–15.83)	(− 0.26 to 0.66)
Eastern Sub-Saharan Africa	2,059		1.23	0.39	10,327	6.45	-0.10	55,541	30.11	0.35
	(1,252-2,823)		(0.77–1.68)	(0.03 to 1.20)	(8,931 − 12,361)	(5.60–7.67)	(− 0.30 to 0.13)	(33,194 − 77,262)	(18.31–41.16)	(− 0.03 to 1.16)
Southern Sub-Saharan Africa	1,351		2.30	0.54	10,865	19.08	0.11	34,716	55.55	0.49
	(890–1639)		(1.51–2.77)	(0.23 to 0.83)	(9,830 − 12,064)	(17.33–21.14)	(− 0.06 to 0.33)	(23,139 − 42,583)	(36.80-67.46)	(0.18 to 0.76)
Western Sub-Saharan Africa	895		0.48	0.84	7,690	4.22	0.18	22,955	11.10	0.78
	(366-1,315)		(0.20–0.69)	(0.16 to 1.69)	(6,369-9,208)	(3.56-5.00)	(− 0.02 to 0.46)	(9,456 − 33,911)	(4.68–16.12)	(0.10 to 1.61)
Southeast Asia, east Asia, and Oceania										
East Asia	18,188		0.83	2.84	13,624	0.63	2.05	354,332	16.31	2.04
	(11,881 − 23,583)		(0.54–1.07)	(0.63 to 5.39)	(9,018 − 17,739)	(0.41–0.81)	(0.27 to 4.12)	(228,713 − 464,089)	(10.44–21.37)	(0.29 to 4.09)
Oceania	26		0.36	0.09	23	0.34	0.07	697	8.43	0.09
	(14–36)		(0.21–0.50)	(− 0.13 to 0.42)	(13–34)	(0.20–0.49)	( -0.14 to 0.39)	(384-1,013)	(4.77–12.17)	(− 0.14 to 0.42)
South-East Asia	16,378		0.91	0.77	14,969	0.86	0.66	387,028	20.51	0.59
	(13,585 − 23,209)		(0.75–1.29)	(0.35 to 1.88)	(12,439 − 21,173)	(0.71–1.21)	(0.26 to 1.71)	(320,924 − 546,241)	(17.00-29.05)	(0.20 to 1.61)

Bold indicates global data and does not denote statistical significance

**Fig. 1:**
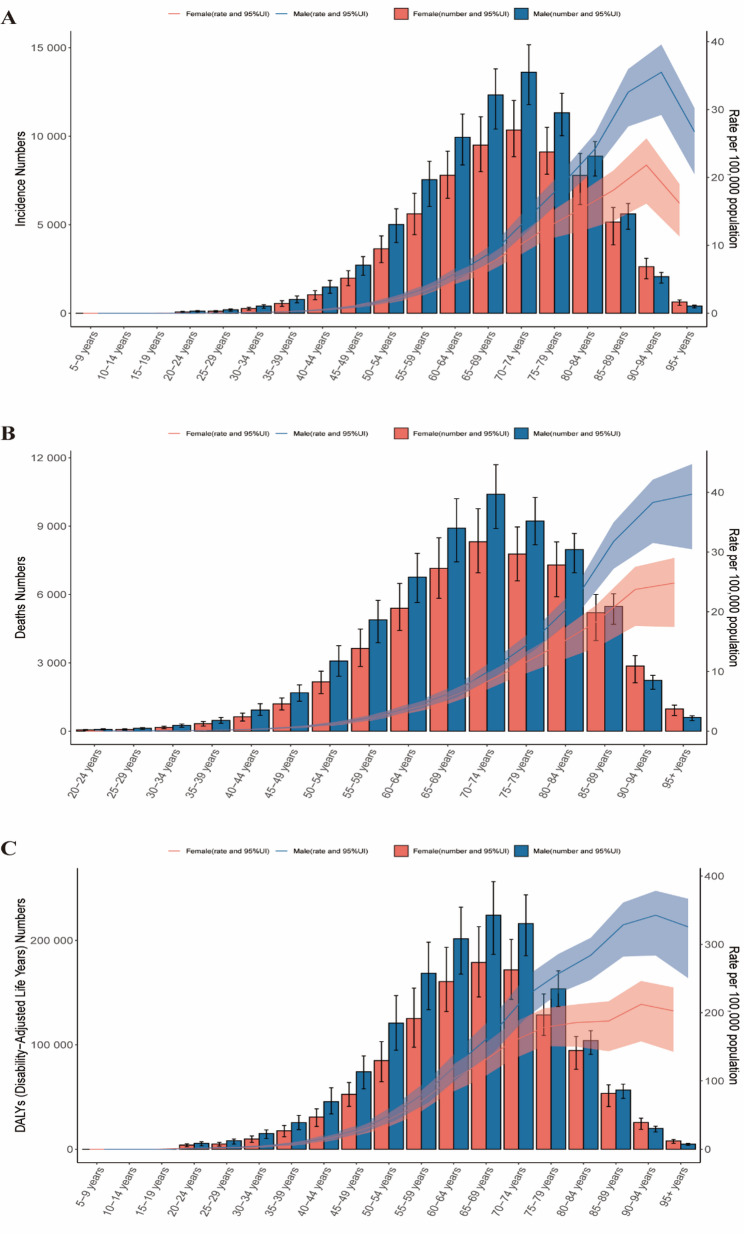
2021 multiple myeloma burden distribution across age cohorts. **A** Incident numbers and age-standardized incidence rate (per 100,000). **B** Death numbers and age-standardized mortality rate (per 100,000). **C** DALYs numbers and age-standardized DALYs rate (per 100,000)

Substantial differences in multiple myeloma burden were observed among various age groups and sexes, with the 75–79 age group exhibiting the highest ASIR and ASMR, contrasting with the 65–69 age group displaying the greatest AS-DALYsR. The disease burden remained persistently greater in males compared to females throughout all age brackets (Fig. 1).

**Fig. 2:**
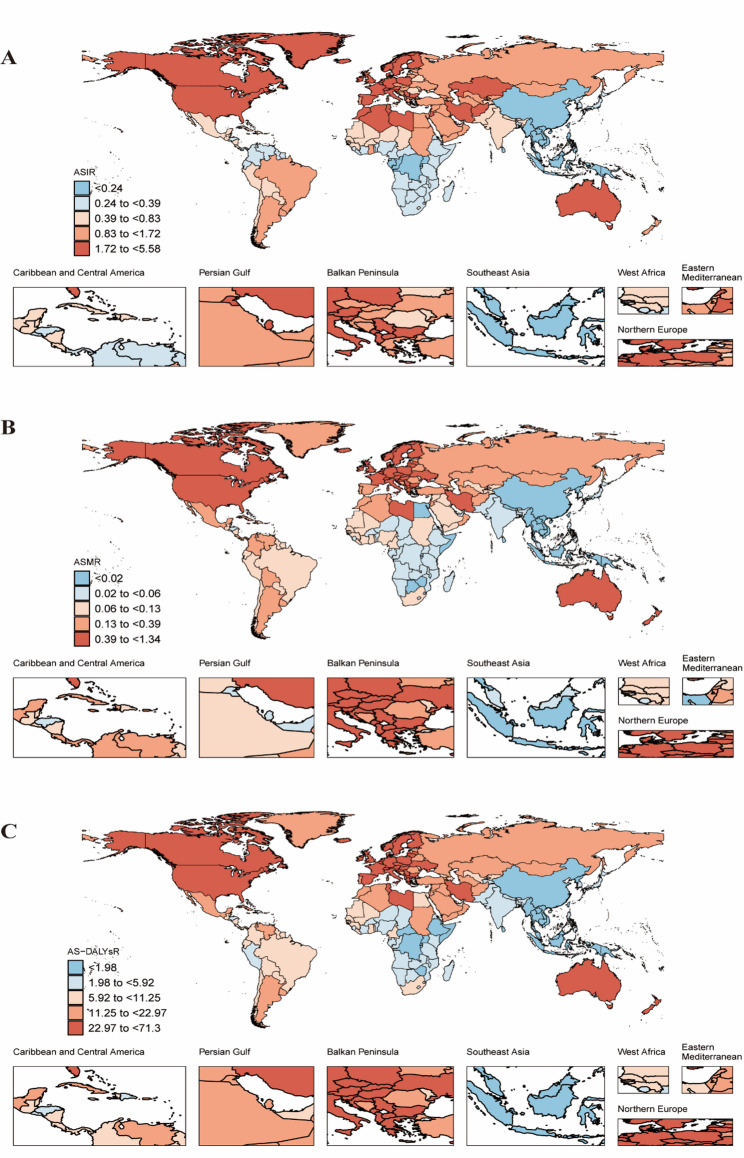
Global age-standardized rates ASR of MM in both sexes combined among 204 countries. **A** Incidence rate, (**B**) mortality, and (**C**) DALYs

Global analysis revealed High-income Australasia as the region with the highest ASIR (5.48 cases per 100,000, 95% UI: 4.77–6.21), while Sweden showed the highest national rate (5.58 cases per 100,000, 95% UI: 4.91–6.34) among 204 countries (Table 1, Table S1, Fig. 2A). The highest regional mortality burden was observed in High-income North America (28.92 cases per 100,000, 95% UI: 26.73–30.38), while the United Kingdom had the highest national ASMR (1.34 cases per 100,000, 95% UI: 1.27–1.40) (Table 1, Table S2, Fig. 2B). The maximal AS-DALYs was observed in High-income Australasia at the regional level (60.65 cases per 100,000, 95% UI: 54.09–67.37), whereas the United Kingdom had the highest national AS-DALYs (71.30 cases per 100,000, 95% UI: 61.83–80.79) (Table 1, Table S3, Fig. 2C). In the five SDI regions, High SDI regions consistently demonstrated the most elevated age-standardized rates for: ASIR, ASMR, and AS-DALYs, with rates of 3.16 cases per 100,000 (95% UI: 2.87–3.34), 2.28 cases per 100,000 (95% UI: 2.05–2.41), and 47.33 cases per 100,000 (95% UI: 44.00–49.82), respectively (Table 1).

### Trends in multiple myeloma disease burden, 1990–2021

Between 1990 and 2021, global MM burden demonstrated a consistent upward trajectory. ASIR exhibited the steepest rise, increasing by 0.18 cases per 100,000 (95% UI: 0.08–0.28). Both the ASMR and the AS-DALYsR increased by 0.06 cases per 100,000 population. By age group, the ASIR, ASMR, and AS-DALYs rates were highest within the 80–94 age group, and all showed an upward trend (Fig. S1-S3).

Within the SDI regions, the most significant increase in the ASIR was noted in the low-middle SDI region, registering an increment of 0.72 cases per 100,000 (95% UI: 0.38–1.52). The High SDI region witnessed the most pronounced elevation in the ASMR, experiencing a rise of 0.72 cases per 100,000 population (95% UI: 2.05–2.41). The Middle SDI region saw the most significant increase in the AS-DALYs, registering an increment of 0.78 cases per 100,000 population (95% UI: 0.37–1.16) (Table 1). Fig. S4 illustrates the annual trend of the ASR of MM among the global population and within each of the five SDI regions spanning the period from 1990 to 2021.

Among the 21 GBD regions, East Asia exhibited the most significant increases in ASIR, ASMR, and AS-DALYs, with increases of 2.84 cases per 100,000 (95% UI: 0.63–5.39), 2.05 cases per 100,000 (95% UI: 0.27–4.12), and 2.04 cases per 100,000 (95% UI: 0.29–4.09), respectively.

**Fig. 3:**
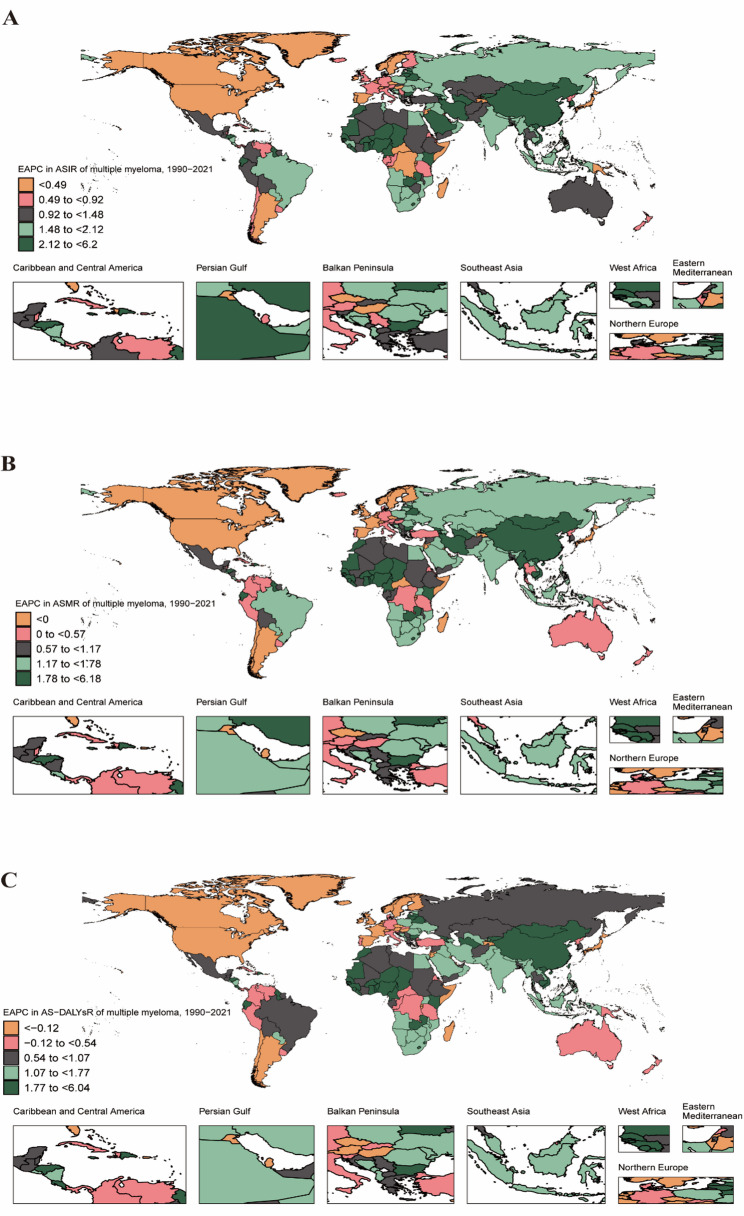
Estimated annual ASR for MM from 1990 to 2021, integrating data of both genders from 204 countries. **A** ASIR, (**B**) ASMR, (**C**) AS-DALYsR

Among the 204 countries, the greatest rise in ASIR was noted in Georgia (EAPC = 6.20, 95% CI: 5.45–6.96) (Table S4, Fig. 3A). The greatest rise in ASMR was also seen in Georgia (EAPC = 6.18, 95% CI: 5.43–6.95) (Table S5, Fig. 3B). The greatest rise in AS-DALYs was identified in Turkmenistan (EAPC = 6.04, 95% CI: 5.45–6.64) (Table S6, Fig. 3C).

### Joinpoint analysis: trends in the disease burden of multiple myeloma across global and SDI regions

**Fig. 4:**
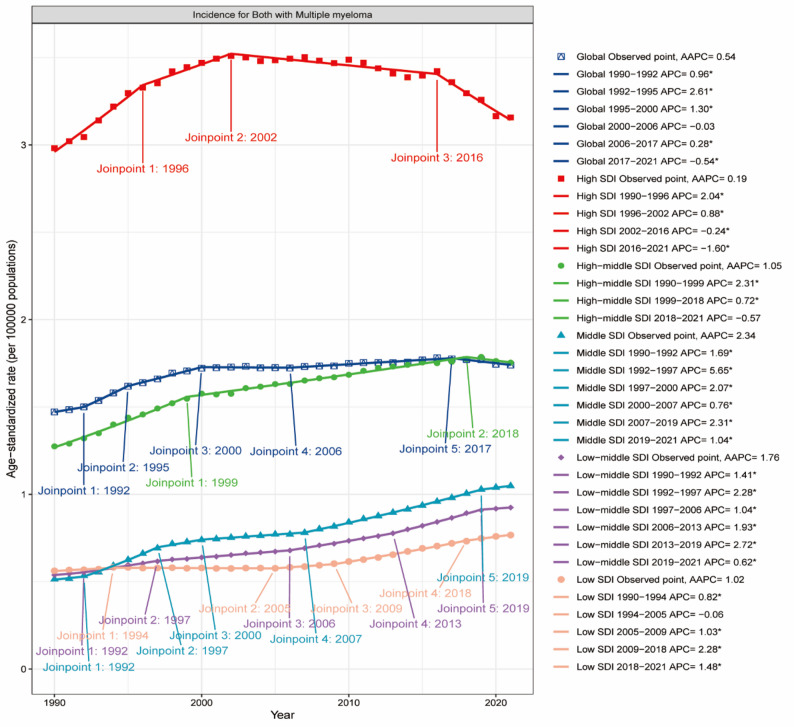
Temporal trends in ASIR for MM on a global scale and within diverse SDI regions (1990–2021), analyzed using the Joinpoint regression model. *p < 0.05

**Fig. 5:**
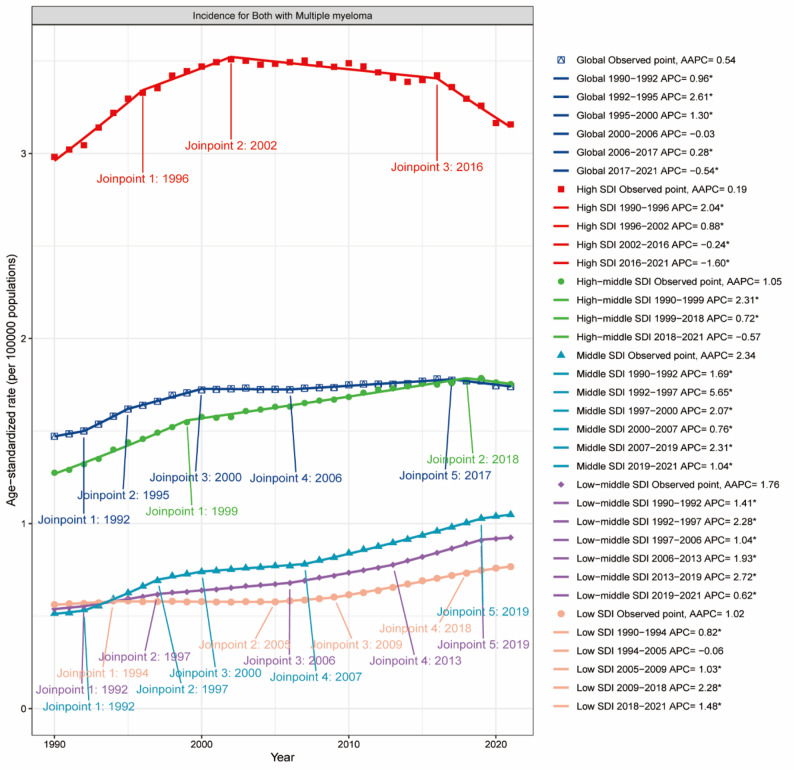
Temporal trends in ASMR for MM on a global scale and within diverse SDI regions (1990–2021)

**Fig. 6:**
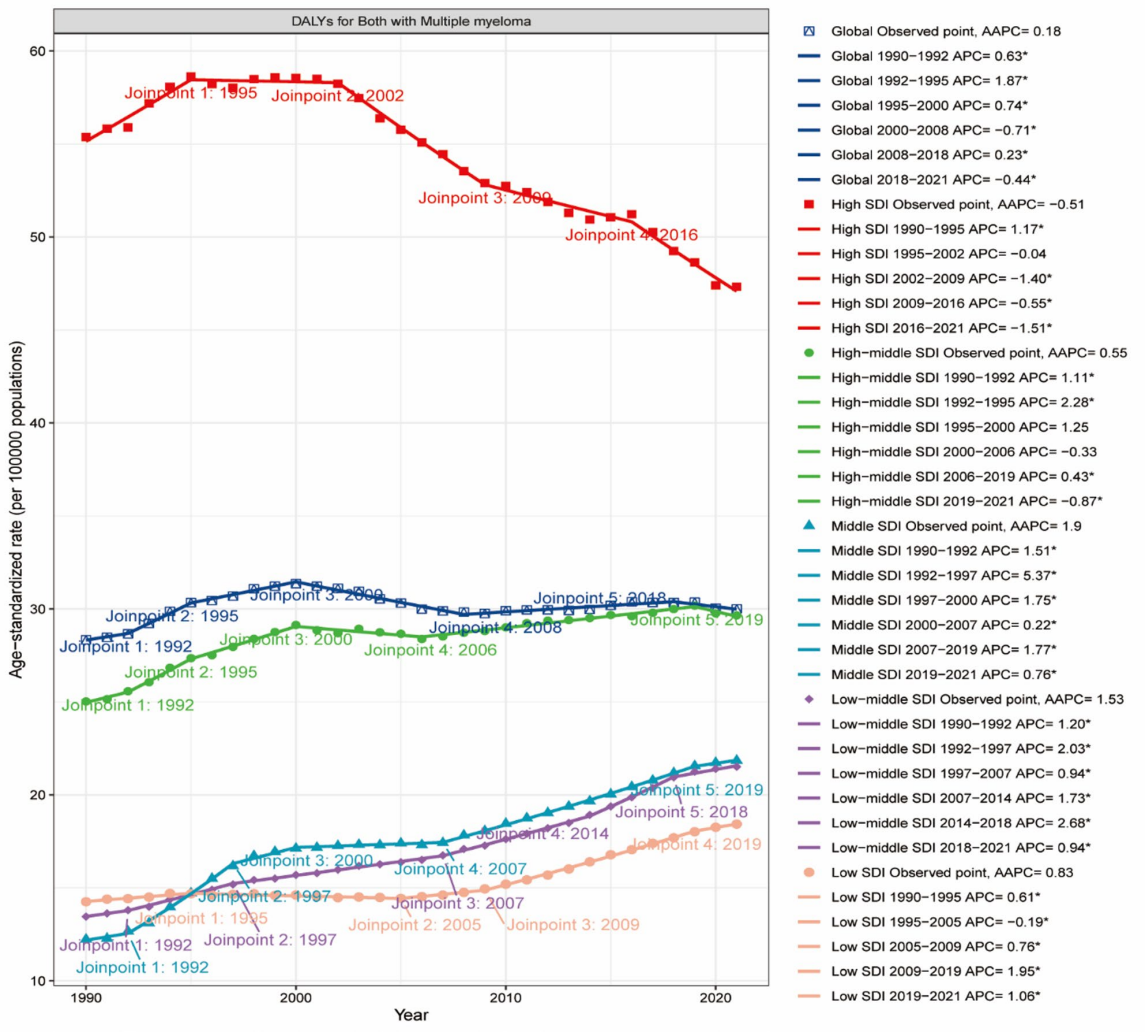
Temporal trends in AS-DALYsR for MM on a global scale and within diverse SDI regions (1990–2021)

The Joinpoint regression model was utilized to analyze MM’s age-standardized rates (including ASIR, ASMR, and AS-DALYsR) across global and within diverse SDI regions during 1990–2021. Globally, MM’s ASIR showed a sustained rise between 1990 and 2021 according to the results (AAPC = 0.54, 95% CI: 0.52–0.57). Among the five SDI regions, the Middle SDI region exhibited the most significant ASIR growth (AAPC = 2.34, 95% CI: 2.31–2.38) (Fig. 4). The global ASMR also showed an overall upward tendency (AAPC = 0.20, 95% CI: 0.17–0.23). Regionally, the Middle SDI regions showed the most remarkable increase in ASMR (AAPC = 1.91, 95% CI: 1.87–1.95) (APC = 5.36, 95% CI: 5.16–5.58) (Fig. 5). The AS-DALYsR rate for MM exhibited a sustained upward trend (AAPC = 0.18, 95% CI: 0.16–0.21). the AS-DALYsR grew most substantially in the Middle SDI region compared to other SDI regions (AAPC = 1.90, 95% CI: 1.87–1.94) (Fig. 6). The trends in ASIR, ASMR, and AS-DALYsR for the other SDI regions (such as High SDI, Low-middle SDI, etc.) are provided in the supplementary materials (Table S7, S8).

### Correlation analysis of ASR, EAPC, and SDI

To investigate the relationship between the MM disease burden and the SDI, we conducted a systematic analysis spanning the years 1990, 2021, and the period from 1990 to 2021. The focus was on examining the correlation between ASIR, ASMR, AS-DALYsR, and the EAPC.

#### Analysis for 1990

In 1990, remarkable negative correlations nationwide were detected between the EAPC and MM disease burden indicators.

**Fig. 7:**
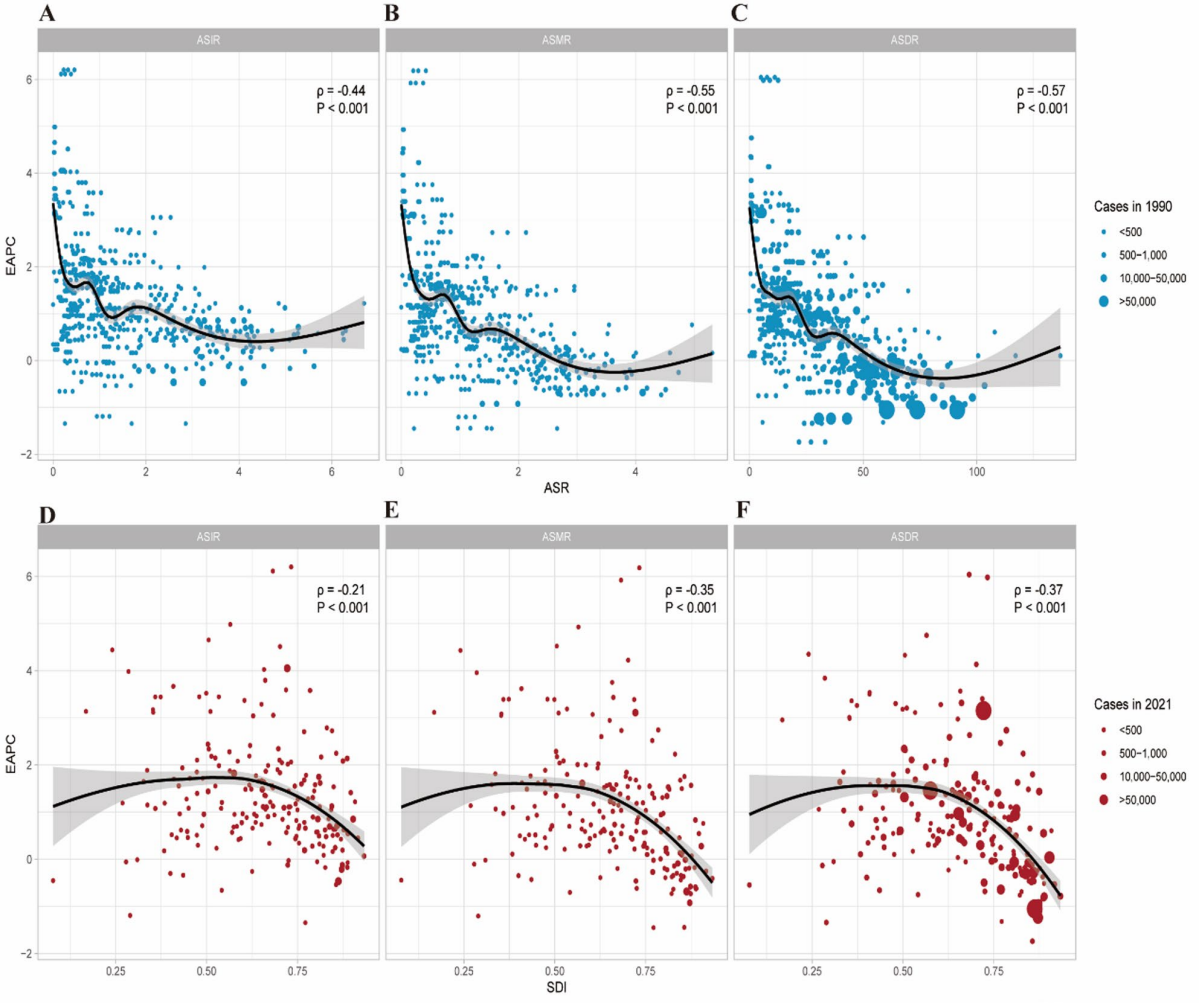
Correlation analyses between EAPCs, ASRs in 1990, and the SDI in 2021. **A** Correlation between EAPCs and ASIR in 1990. **B** Correlation between EAPCs and ASMR in 1990. **C** Correlation between EAPCs and AS-DALYsR in 1990. **D** Correlation between EAPCs and SDI in 2021 for ASIR. **E** Association between EAPCs and SDI in 2021 for ASMR. **F** Association between EAPCs and SDI in 2021 for AS-DALYsR. Each circle corresponds to one of the 204 nations or regions, with the circle size proportional to the number of MM patients. ρ: Pearson’s correlation coefficient

- ASIR: ρ=-0.44, with a P-value less than 0.01.
- ASMR: ρ=-0.55, with a P-value less than 0.01.
- AS-DALYsR: ρ=-0.57, with a P-value less than 0.01.
- In this context, ρ denotes the correlation coefficient, where associations with *P* < 0.05 were considered statistically significant. These results suggest that in countries with higher SDI, the rate of increase in MM disease burden was relatively slower, while in lower SDI countries, the growth rate was faster (Table S9, Fig. 7A-C).

#### Analysis for 2021

By 2021, the EAPC and the ASR for SDI regions again demonstrated a significant negative correlation.

**Fig. 8:**
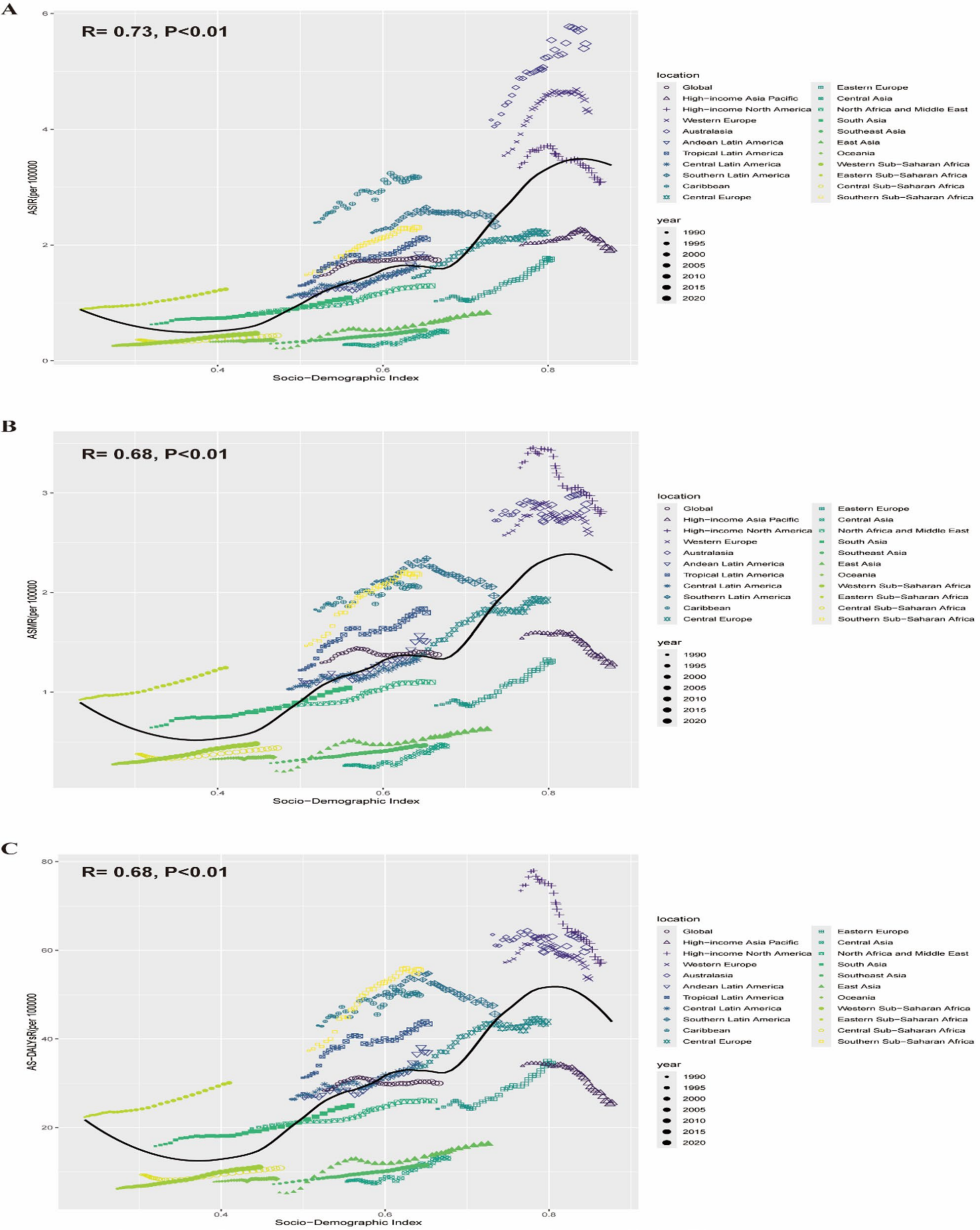
Trends and correlation of ASRs with SDI from 1990–2021. **A** ASIR and SDI trends and their relationship in 22 regions. **B** ASMR and SDI trends and their relationship in 22 regions. **C** AS-DALYsR and SDI trends and their relationship in 22 regions

- ASIR: ρ=-0.21, with a P-value less than 0.01.
- ASMR: ρ=-0.35, with a P-value less than 0.01.
- AS-DALYsR: ρ=-0.37, with a P-value less than 0.01.
- This indicates that while the MM disease burden growth rate slowed in high SDI regions, the overall burden remained relatively high (Table S9, Fig. 8D, E, F).

#### Analysis for 1990–2021

During the 1990–2021 period, significant positive associations emerged between MM’s ASIR, ASMR, and AS-DALYsR with SDI.

- ASIR: *R* = 0.73, with a P-value less than 0.01.
- ASMR: *R* = 0.68, with a P-value less than 0.01.
- AS-DALYsR: *R* = 0.68, with a P-value less than 0.01.

In high-income regions (High-income Australasia, Western Europe, High-income North America), these indicators were substantially higher than the global average (Table S10-12, Fig. S5, Fig. 8A-C). This suggests that despite abundant healthcare resources in these regions, the diagnostic and treatment burden for MM remains substantial.

### Analysis of risk factors

To assess the contribution of high BMI to MM’s disease burden, a comprehensive evaluation was carried out by us regarding the ratio of MM-related deaths and DALYs resulting from high BMI both globally and in different regions. The results showed significant regional variations in the disease burden stemming from high BMI. The regions with the highest proportions were primarily located in high SDI areas, including High-income Australasia, Southern Latin America, and High-income North America, where deaths accounted for 10.2%, 10.6%, and 11%, respectively. The proportions of DALYs were 10.6%, 11%, and 11.5%, respectively. On the contrary, the regions having the lowest proportions were concentrated in low-middle SDI areas, including South Asia, High-income Asia Pacific, and Southeast Asia, where deaths accounted for 3.3%, 4%, and 4.1%, respectively. The proportions of DALYs were 3.6%, 4.3%, and 4.4%, respectively.

**Fig. 9:**
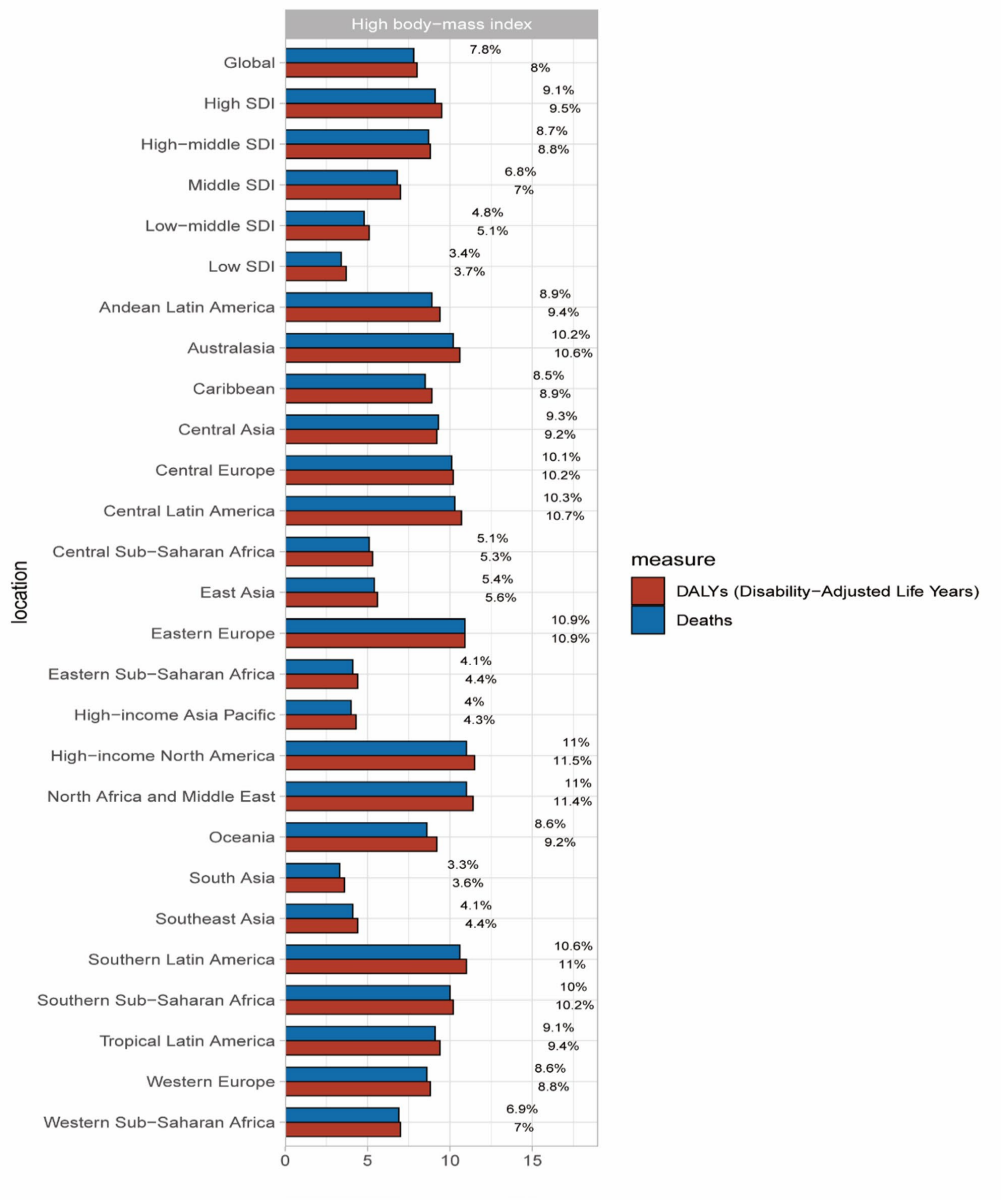
The ratio of global MM disease burden resulting from high BMI, among GBD regions, and the five SDI regions

Trend analysis across SDI regions revealed that the proportions of MM burden stemming from high BMI in diverse SDI regions were consistent with the regional findings mentioned above. The MM burden attributable to high BMI showed markedly greater values in high SDI areas than in low SDI regions, and the trend of this burden followed a similar pattern to that of SDI regional distribution (Fig. 9).

In conclusion, the influence of high BMI regarding MM’s disease burden exhibits notable regional disparities, with a significantly higher contribution in high-income regions. This highlights that high BMI, being a crucial risk factor for MM, requires more attention in high-income regions, while in low-income regions, a more comprehensive approach to other health burdens and intervention strategies is needed to optimize disease management and prevention globally.

### MR analysis

**Fig. 10:**
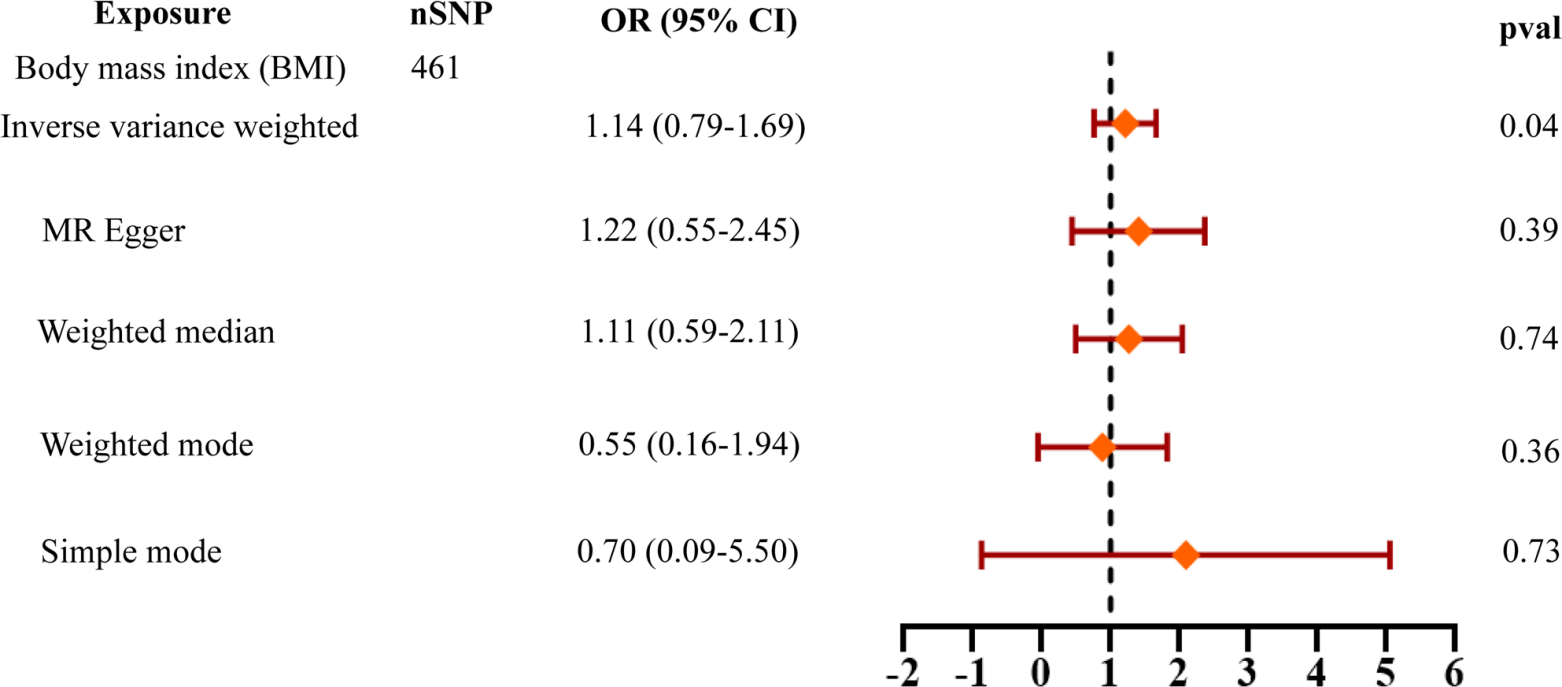
Forest plot of Mendelian randomization (MR) analysis of the BMI-MM risk relationship

We conducted an MR analysis, primarily utilizing the inverse-variance weighted (IVW) method to assess BMI-MM causality [26]. Following rigorous selection, 513 SNPs significantly associated with BMI were included in our two-sample MR analysis. These genetic instruments demonstrated F-statistics ranging from 28.6 to 1426.1 (Table S14), confirming their strength as valid IVs. The findings from the IVW approach indicated that a rise in BMI was linked to an increased risk of MM (OR, 1.14; 95% CI, 0.79–1.69; *P* = 0.04) (Fig. 10). No confounding with outlier SNPs was found before MR analysis. Heterogeneity tests suggested no heterogeneity (P_*Q*-test_ = 0.99). There was no pleiotropy in the MR Egger test (P_intercept_ = 0.21). Funnel plot and scatter plot demonstrated no heterogeneity as well as horizontal pleiotropy in the MR analysis of BMI versus MM (Fig. S6A-B). The leave-one-out method confirmed the robustness of the causal association, as no SNP exerted significant influence when individually excluded (Fig. S6C). It has been reported that lower plasma adiponectin concentrations show a significant association with increased MM risk among overweight and obese populations [27]. Lipocalin, an important cytokine secreted by adipose tissue, whose expression level is negatively related to BMI, has been demonstrated to impede the proliferation of MM cells and decrease tumor angiogenesis [28]. Thus, the downregulation of lipocalin levels induced by elevated BMI may be a key molecular mechanism mediating the risk of obesity-associated MM, further providing evidence for a possible causal link between BMI and MM.

### Future trends in multiple myeloma

**Fig. 11:**
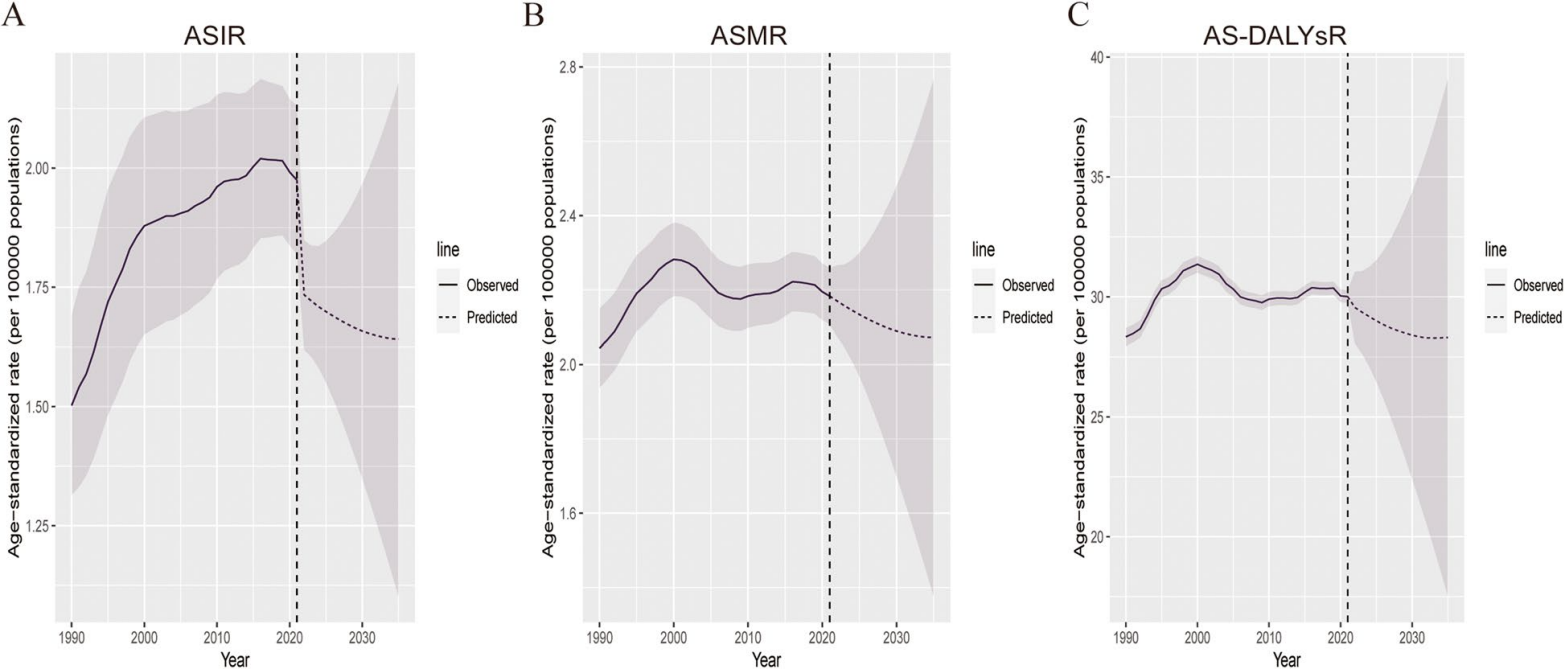
Predicted trends for multiple myeloma (MM) disease burden indicators from 2021–2035. **A** Projected ASIR (incidence per 100,000 population). **B** Projected ASMR (mortality per 100,000 population). **C** Projected AS-DALYsR (disability-adjusted life years per 100,000 population)

To further improve the prevention strategies for MM, this investigation employed the BAPC model to forecast the disease burden trends globally through 2035, focusing on three key indicators: ASIR, ASMR, and AS-DALYsR. The results are as follows:

1. *Global Trends*: It is anticipated that the global ASIR for MM will decrease. By 2035, the global ASIR is projected to be 1.64 cases per 100,000 population (95% CI: 1.10–2.18) (Fig. 11A). The ASIR for men is projected to be 1.98 cases per 100,000 (95% CI: 1.34–2.61), while the ASIR for women is expected to be 1.31 cases per 100,000 (95% CI: 0.89–1.75) (Table S13).
2. *Mortality Trends*: The ASMR for MM is also expected to decline. By 2035, the global ASMR is forecasted to be 2.07 cases per 100,000 (95% CI: 1.38–2.77) (Fig. 11B). The ASMR for men is projected to be 2.50 cases per 100,000 (95% CI: 1.71–3.29), while for women, the ASMR is expected to be 1.67 cases per 100,000 (95% CI: 1.10–2.25) (Table S13).
3. *Impact on Population Health*: The overall impact of MM on population health is expected to gradually decrease, as reflected in the continuous decline in the AS-DALYsR. Projections indicate that the global AS-DALYs will attain 28.30 per 100,000 population (95% CI: 17.52–39.08) by 2035 (Fig. 11C); The disease burden will mainly concentrate in the male group. According to projections, the AS-DALYs rate will reach 33.22 per 100,000 population (95% CI: 20.81–45.63) among males by 2035, compared to 23.03 per 100,000 (95% CI: 14.47–31.59) in females (Table S13).

Projection analyses suggest that while the incidence, mortality, and DALYs rates of MM are projected to decrease, the disease burden for men remains significantly higher than for women. Future global prevention and control strategies should prioritize early screening and intervention for the male population. Moreover, achieving these declining trends will necessitate sustained efforts to enhance global health education, optimize healthcare services, and strengthen disease surveillance.

## Discussion

This study represents the first comprehensive and systematic analysis of the global disease burden of multiple myeloma (MM). By integrating MR with the GBD dataset, it examined trends of age-ASIR, ASMR, and AS-DALYsR up to 2035. Additionally, this investigation specifically examined variations in disease burden across different SDI regions and age demographics. Based on these analyses, we comprehensively examined the incidence, deaths, and DALYs linked to MM, aiming at providing scientific evidence to support the development of public health strategies for MM in nations worldwide.

First, significant regional disparities existed in the global disease burden. In 2021, the ASIR, ASMR, and AS-DALYsR for MM were highest in high-income regions, which were primarily attributed to higher disease detection rates and more robust health statistics systems. Conversely, low-income regions, particularly Central Sub-Saharan Africa and Oceania, had the lowest disease burden. This may be attributed to limited healthcare resources and insufficient statistical data, which likely underestimate the true burden of the disease [29, 30]. These results indicate potential underestimation of MM burden in low-income areas, and further evaluation needs to be conducted, considering local healthcare resources.

Between 1990 and 2021, there were considerable differences in the trends and driving factors of MM among global and SDI regions. The ASIR showed a slow upward trend both globally and across the five SDI regions, largely as a result of population growth, aging, and advancements in diagnostic technologies [29, 31–33]. Despite the increase in ASIR, the ASMR exhibited a downward trend globally and in high-SDI regions, largely due to improvements in treatment options and healthcare standards. The decline in MM mortality rates in High-income regions, including North America and Eastern Europe, reflects the effectiveness of medical interventions [34, 35]. However, in regions with significant disparities in access to and availability of treatments, such as Central Asia, mortality rates have instead risen [36]. These regional differences highlight that the distribution of healthcare resources and access to advanced treatment technologies are key factors influencing the disease burden of MM.

Moreover, due to differences in national and population characteristics, the disease burden between sexes follows a similar pattern to the overall disease burden, with men experiencing a higher burden than women, which corresponds to what was discovered in prior research. This disparity might be associated with genetic differences and variations in exposure to risk factors [37]. On a national scale, between 1990 and 2021, Georgia exhibited the fastest growth in ASIR (EAPC = 6.20, 95% CI: 5.45–6.96), which may be associated with the increase in per capita GDP across countries [38]. In contrast, for high SDI countries, both the ASMR and DALY rates have demonstrated a downward trend, strongly associated with their advanced prevention and healthcare systems as well as effective preventive strategies [39, 40]. For example, the reduction in MM mortality rates and DALY rates in countries like the United States and Japan has been extremely pronounced. A recent study indicates that these countries rank among the highest globally in healthcare indices [41].

From an age perspective, individuals aged 70 to 95 are the primary population burdened by MM. As an age-related disease, the incidence of MM correlates positively with age, which results in the elderly constituting the majority of MM patients. It is projected that by 2035, 77% of newly diagnosed MM patients will be over the age of 65 [42, 43]. Despite advancements in treatment that have extended patient survival, the improvement in older patients is less pronounced compared to younger patients. This may be attributed to poorer treatment tolerance and the adverse effects of combination therapies in the elderly [44–47]. Therefore, developing personalized treatment plans for older patients is essential for improving MM’s global disease burden.

The Joinpoint regression analysis revealed distinct temporal patterns in the MM burden across different SDI regions. During 1990–2021, Middle SDI regions showed the most rapid increase in MM burden, whereas High SDI regions experienced a marked decline. The increasing trend in Middle SDI regions may be explained by population growth, population aging, improved disease detection, and a gradual increase in exposure to lifestyle-related risk factors, such as high BMI. However, limited access to early diagnosis, novel therapies, and standardized long-term care may have further contributed to the rising mortality and DALY burden in these regions. In contrast, the decreasing burden observed in High SDI regions is likely associated with well-established healthcare systems, earlier diagnosis, wider availability of effective treatments, improved supportive care, and better disease management. As SDI levels increase and diagnostic capacity and healthcare resources improve, the ASR of MM may follow a “rise-then-decline” pattern, reflecting an initial increase due to improved case detection, followed by a subsequent decrease driven by advances in treatment and prevention strategies [48, 49]. Nevertheless, the burden may remain high in low- and middle-income regions because of delayed diagnosis, treatment gaps, and unequal access to advanced medical care, highlighting the urgent need to strengthen MM diagnosis, treatment, and care capacity in these areas [50].

Obesity and the metabolic syndrome linked to it have come to the fore as major hurdles in the sphere of global public health [51]. Current epidemiological evidence demonstrates a significant correlation between high BMI levels and greater MM mortality risk among Asian populations [52]. Thus, our investigation carried out an attribution analysis of the MM disease burden, identifying high BMI as a key modifiable risk factor. The burden attributable to high BMI is particularly significant in high SDI regions, especially in areas like High-income Australasia, Southern Latin America, and High-income North America. In addition, this study first applied an MR methodology to explore potential causal relationships between BMI and MM susceptibility. This finding provided a new research direction for understanding the molecular mechanisms of obesity-associated tumor risk and potentially guides the development of targeted prevention strategies. Studies have shown that a persistent upward trend in the global cancer-related mortality attributed to high BMI from 2010 to 2019 [53]. The variation in the disease burden across regions is primarily driven by differences in economic development levels and regional dietary habits. Our findings indicate that high BMI associated with the MM burden presents particularly significant public health implications in high SDI regions. Factors such as high-calorie diets, sedentary lifestyles, and well-established food supply chains have exacerbated obesity problems in high SDI regions, including High-income North America and Western Europe, leading to the highest disease burden of MM [54, 55]. In contrast, this issue is less pronounced in low SDI regions with lower economic levels. However, the high-income population in these low-income countries remains the primary contributor to the regional MM disease burden [56]. These results underscore the importance of promoting healthy diets and lifestyles, particularly in high-income regions.

Finally, the BAPC model predicts a continued declining trend in the global MM disease burden through 2035. The reasons for this projected decline can be explained by several factors. First, continued investment in cancer research and the rapid development of targeted therapies, immunotherapies, and precision medicine are expected to improve survival and reduce mortality and DALYs [57–59]. For example, targeted therapies such as MT-0169, small peptides targeting CD38, CD38-related immunotherapies, and artificial intelligence-assisted diagnostic approaches may contribute to earlier diagnosis, better risk stratification, and improved quality of care for MM patients [60]. Second, improvements in public health policies, including enhanced cancer screening, health education, early referral, and strengthened prevention and control programs, may further reduce disease progression and adverse outcomes. Third, broader access to standardized treatment regimens, supportive care, and follow-up management may help reduce mortality, especially in regions where therapeutic resources are expanding. Nevertheless, the projected decline in age-standardized rates does not necessarily imply a reduction in the absolute number of MM cases, because population growth and aging may continue to increase the number of affected individuals. Therefore, future strategies should focus not only on reducing age-standardized mortality and DALY rates but also on preparing healthcare systems for the growing number of older MM patients.

While this study offers the first comprehensive global evaluation of the burden of MM, its associated risk factors, and projected epidemiological trends through 2035, several methodological limitations should be acknowledged. First, variations in socioeconomic development and medical resources across countries have led to heterogeneity in the quality of GBD data, which may affect the accuracy of estimates. Therefore, the results should be interpreted with caution [61, 62]. Second, given the long study period (1990–2021), certain short-term dynamic changes in disease burden within specific years or countries may not have been fully captured. Third, although the BAPC model offers valuable projections based on historical trends, future estimates could be influenced by unforeseen changes in diagnostic practices, treatment availability, demographic shifts, health policies, and exposure to risk factors. These uncertainties should be carefully considered when interpreting the projected MM burden through 2035.

## Conclusions

In summary, between 1990 and 2021, the global disease burden associated with MM has been on the continuous increase, and the regions with the most significant burden are those with high-income levels. Nations with middle SDI levels have grown more rapidly in disease burden, underscoring the urgent requirement for enhanced oncology care infrastructure and therapeutic interventions in these areas. Meanwhile, the relatively low reported burden in low-income regions should be interpreted with caution, as limited healthcare resources and incomplete disease registration may lead to underestimation of the true burden. Given global population aging, targeted strategies that account for age-specific and region-specific differences are essential for reducing the future burden of MM. Although the BAPC model projected a declining trend in the global age-standardized MM burden through 2035, the absolute number of MM cases may continue to increase due to population growth and aging; therefore, these projections should be interpreted cautiously. Specifically, interventions targeting dietary and lifestyle factors in high-income regions are projected to substantially lower the MM burden. Moving forward, continued advancement in MM-related research and medical technology is essential to further optimize global disease prevention and control systems and reduce the health loss associated with MM.

## Supplementary Information

Supplementary Material 1.

Supplementary Material 2.

## Data Availability

All data used in this study were obtained from the publicly available Global Burden of Disease Study 2021 (GBD 2021), available at https://vizhub.healthdata.org/gbd-results/ .

## Abbreviations

MM: multiple myeloma
DALYs: Disability-adjusted life years
BAPC: Bayesian Age-Period-Cohort
SDI: Socio-Demographic Index
ASIR: Age-standardized incidence rate
ASMR: Age-standardized mortality rate
ASPR: Age-standardized prevalence rates
AAPC: Annual average percentage change
ASRs: Age-standardized rates
EAPC: Estimated Annual Percentage Change
