# Supplementary material for: Global burden of multiple myeloma(1990–2021) and projections up to 2035: a methodical analysis leveraging the global burden of disease 2021 study and Mendelian randomization
Source: J Egypt Natl Canc Inst. 2026 Jul 9;38:44. doi: 10.1186/s43046-026-00377-4 (PMC13350782; doi:10.1186/s43046-026-00377-4)
Supplement: Supplementary file 1 — Supplementary Material 1. [file 43046_2026_377_MOESM1_ESM.docx]

**Supplementary Figures**

**
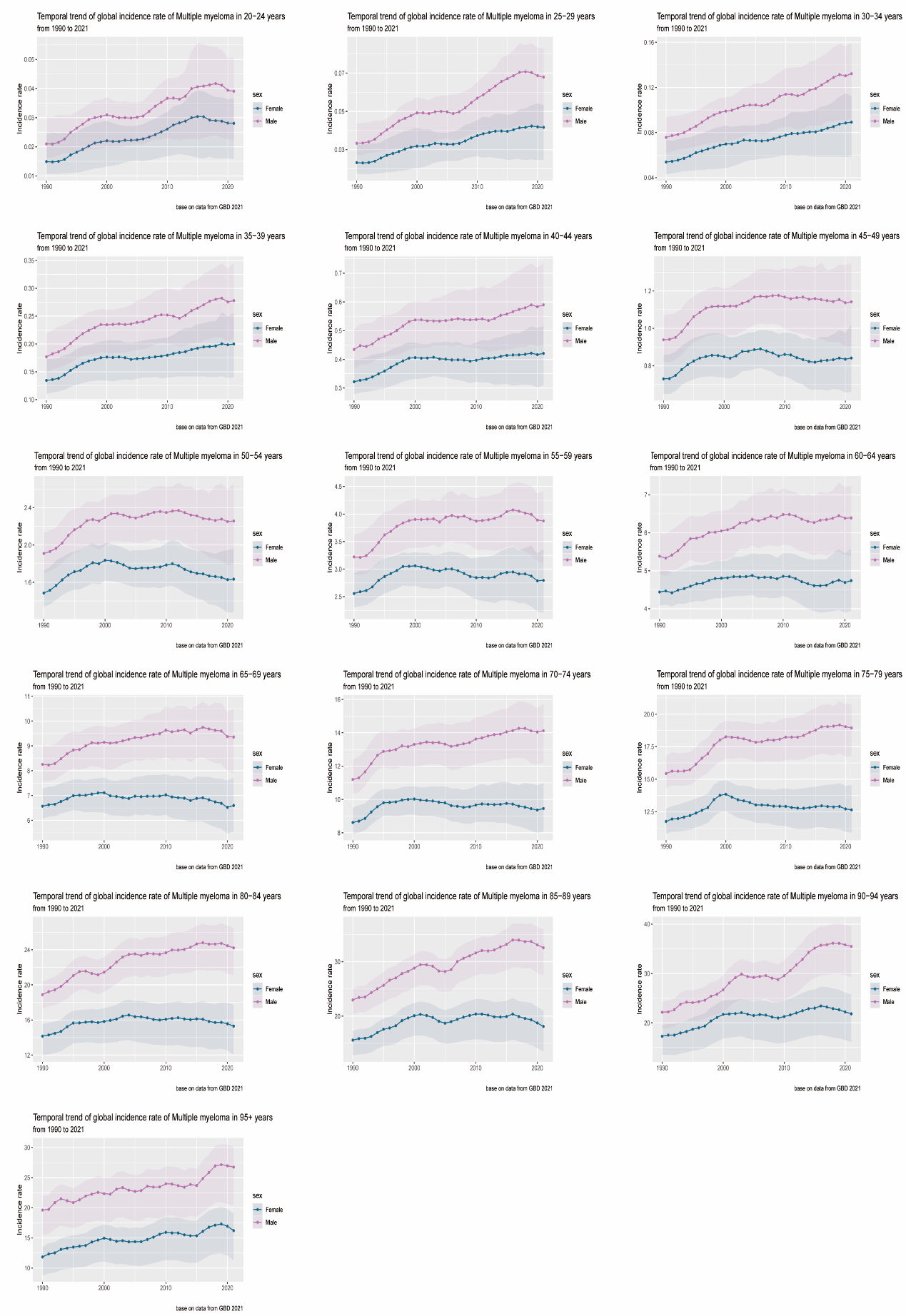
**

S1 Fig. Changes of multiple myeloma ASIR in different age groups and genders from

1990 to 2021.


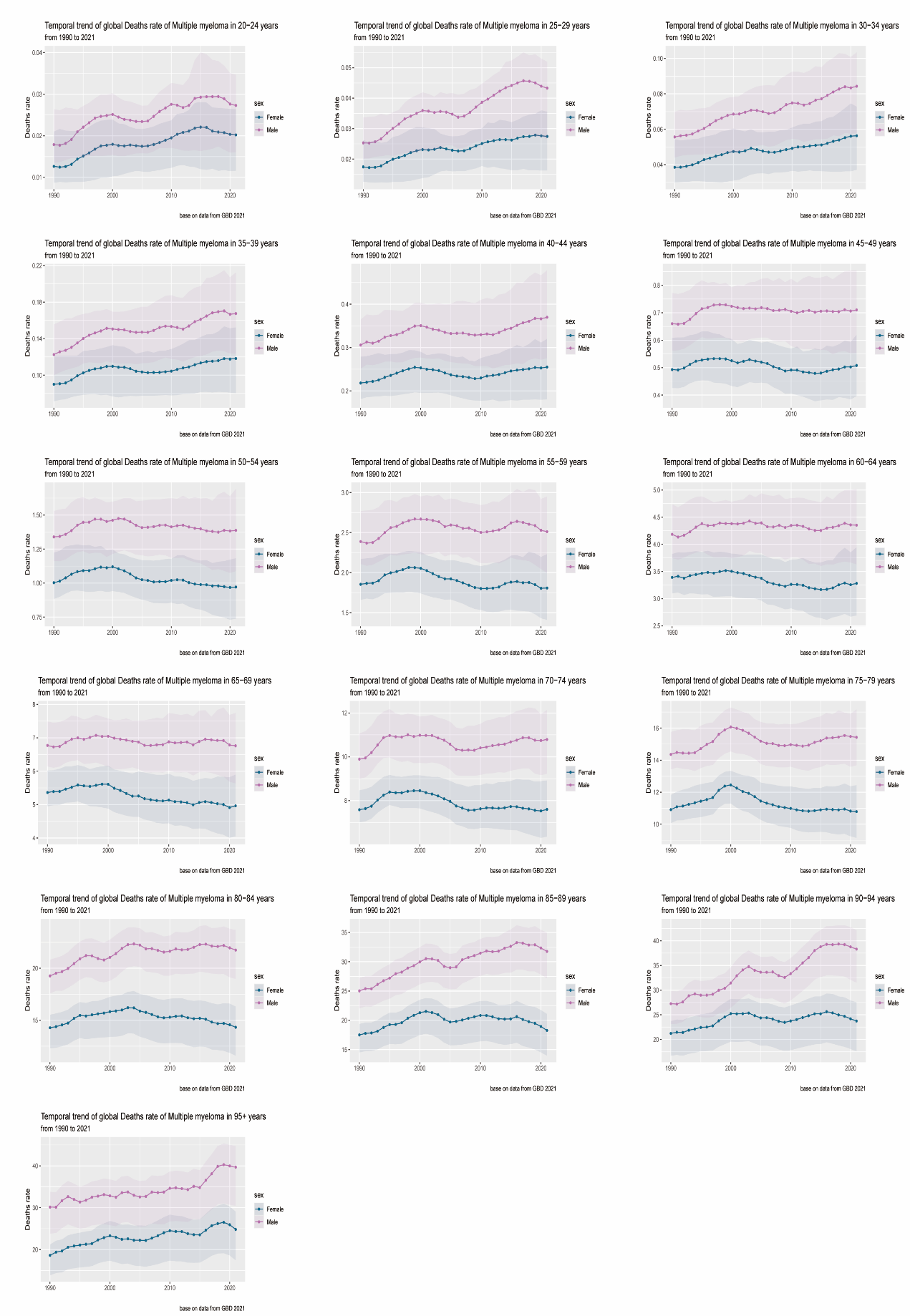


S2 Fig. Changes of multiple myeloma ASMR in different age groups and genders

from 1990 to 2021.


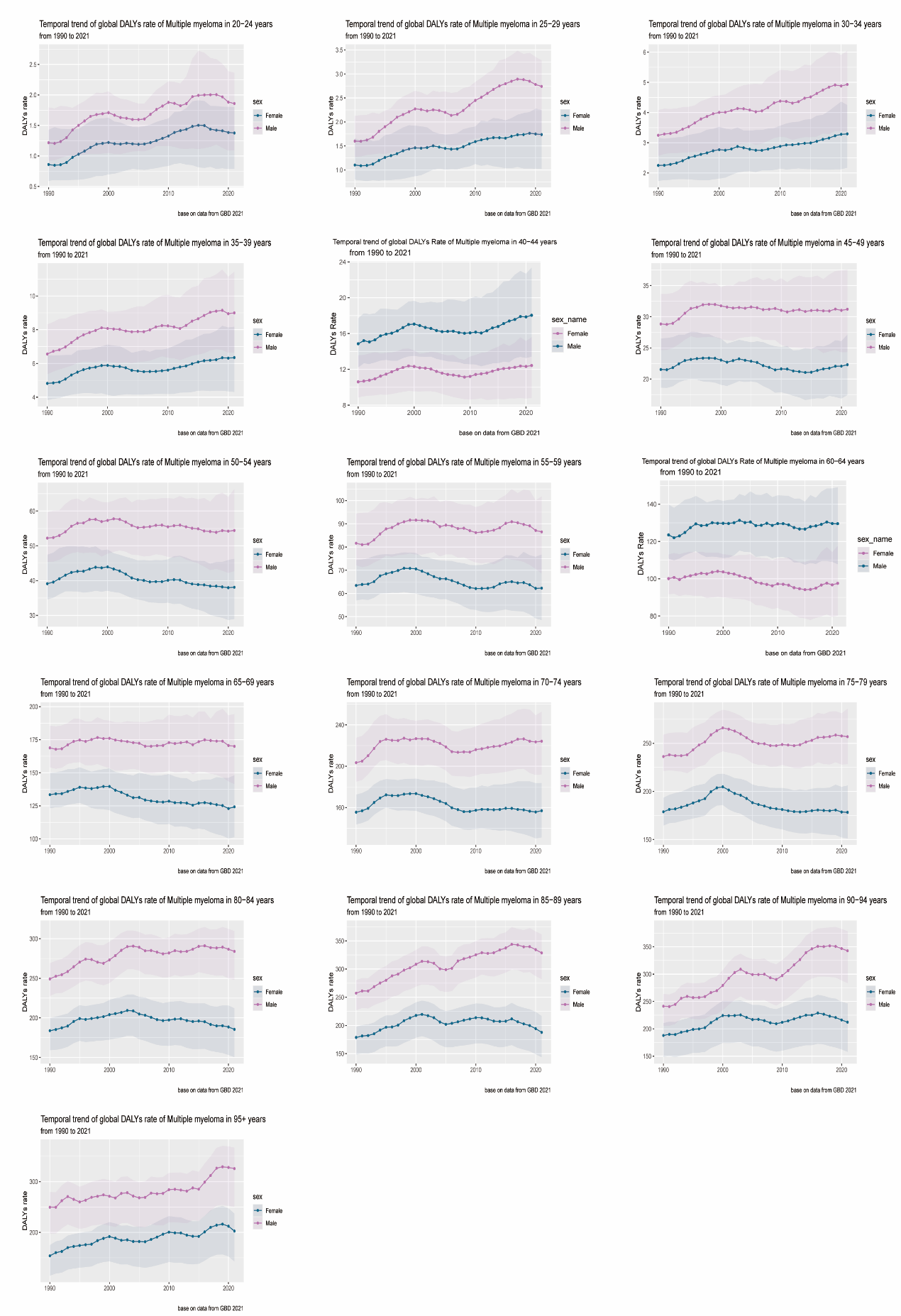


S3 Fig. Changes of multiple myeloma AS-DALYsR in different age groups and

genders from 1990 to 2021.


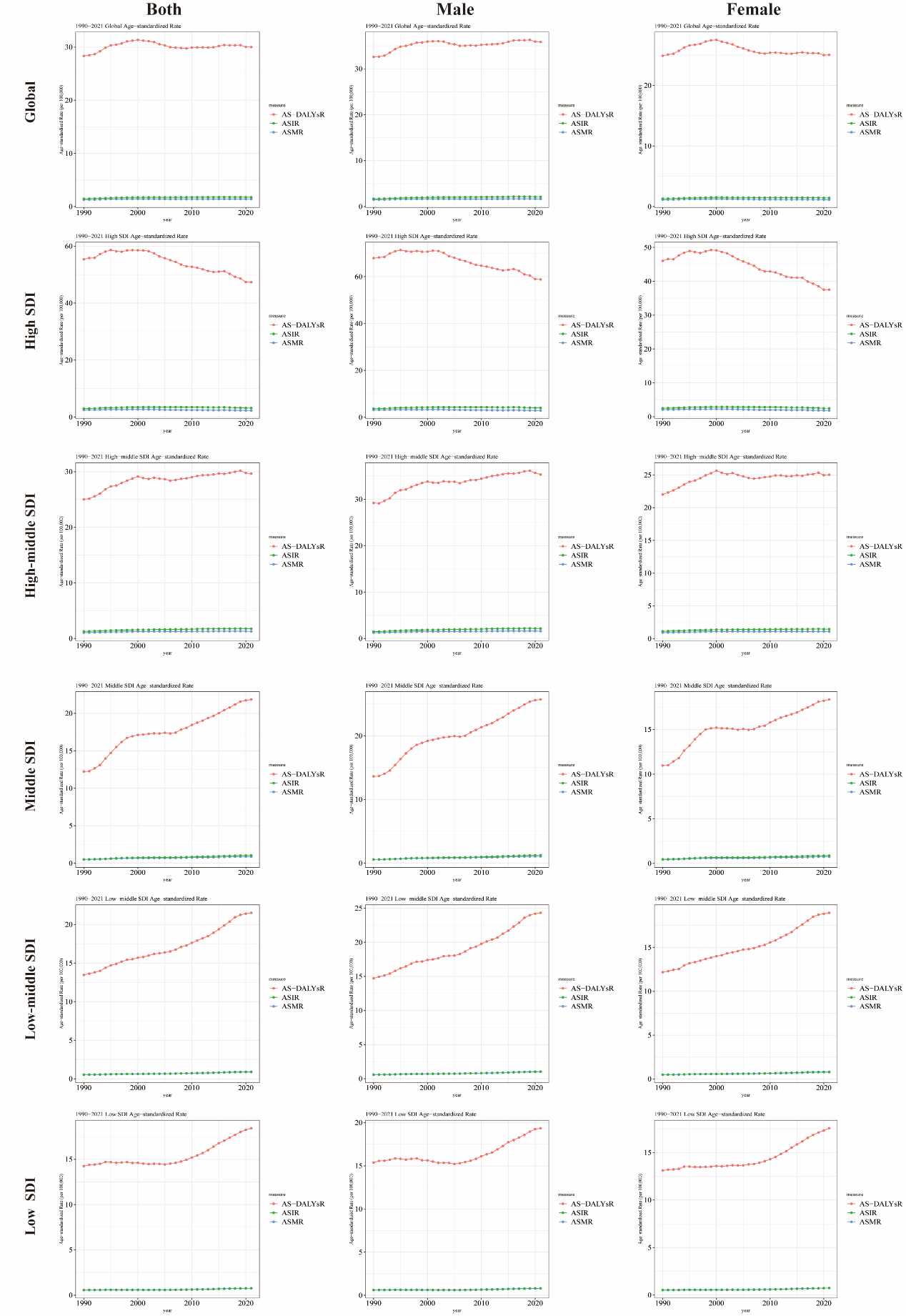


S4 Fig. Trends of ASIR, ASMR, and AS-DALYsR in MM between the sexes from 1990 to 2021.


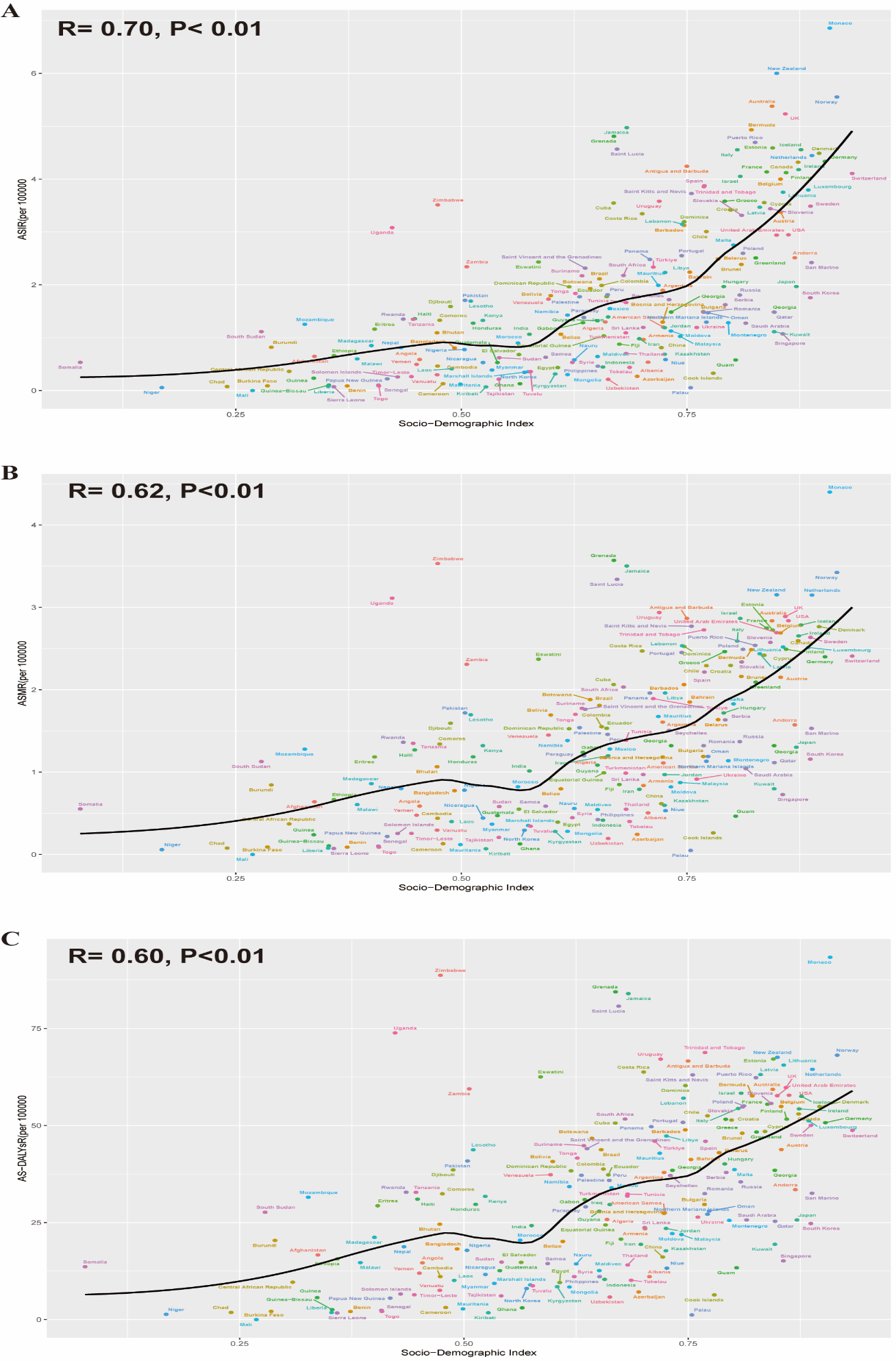


S5 Fig. Correlation analysis of ASRs with 204 countries. (A) ASIR. (B) ASMR. (C) AS-DALYsR.


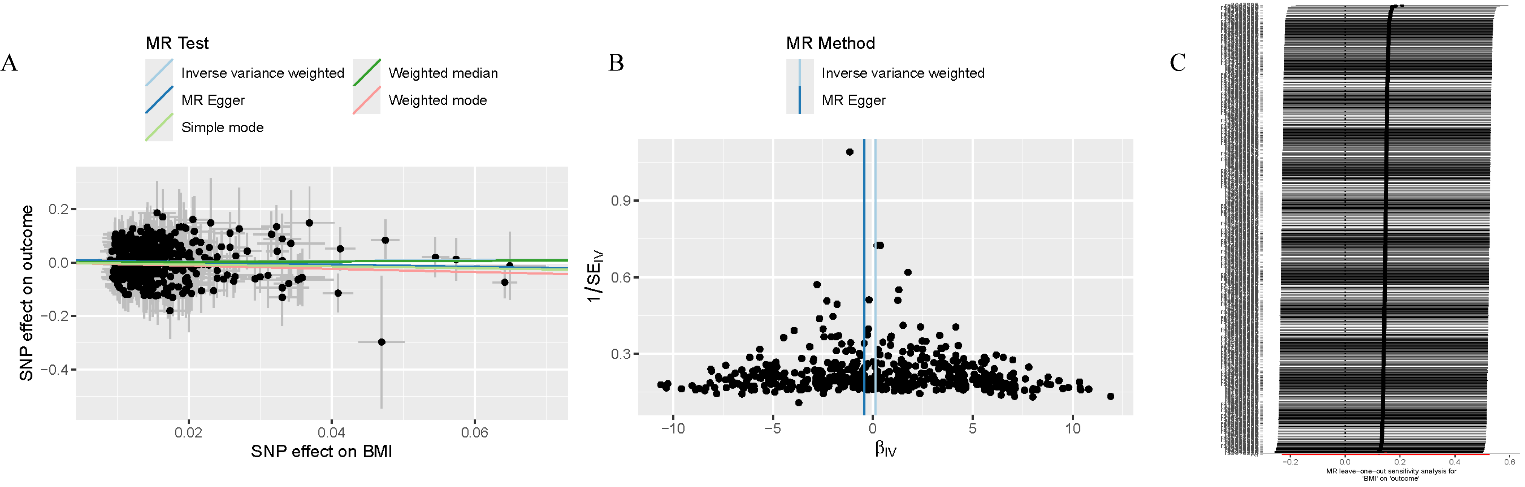


S6 Fig. Results of Mendelian randomization (MR) analysis of BMI and multiple myeloma (MM) risk. (A) MR scatter plot of BMI and MM risk, with the slope of the regression line reflecting the magnitude of the causal effect; (B) Funnel plot for heterogeneity assessment of MR analysis; (C) Leave-one-out sensitivity analysis of BMI on MM risk to verify the robustness of the results.
